# *Escherichia coli* Strains Isolated from the Uteri Horn, Mouth, and Rectum of Bitches Suffering from Pyometra: Virulence Factors, Antimicrobial Susceptibilities, and Clonal Relationships among Strains

**DOI:** 10.1155/2014/979584

**Published:** 2014-03-09

**Authors:** Juliana M. A. Agostinho, Andressa de Souza, Ruben P. Schocken-Iturrino, Lívia G. Beraldo, Clarissa A. Borges, Fernando A. Ávila, José M. Marin

**Affiliations:** ^1^Programa de Microbiologia Agropecuária, Faculdade de Ciências Agrárias e Veterinárias, UNESP-Campus de Jaboticabal, 14884-900 Jaboticabal, SP, Brazil; ^2^Departamento de Medicina Veterinária Preventiva, Faculdade de Ciências Agrárias e Veterinárias, UNESP-Campus de Jaboticabal, 14884-900 Jaboticabal, SP, Brazil; ^3^Departamento de Patologia Veterinária, Faculdade de Ciências Agrárias e Veterinárias, UNESP-Campus de Jaboticabal, 14884-900 Jaboticabal, SP, Brazil; ^4^Departamento de Morfologia, Fisiologia e Patologia Básica, FORP, Avenida do Café S/N, Campus USP, 14040-904 Ribeirão Preto, SP, Brazil

## Abstract

Pyometra is recognized as one of the main causes of disease and death in the bitch, and *Escherichia coli* is the major pathogen associated with this disease. In this study, 70 *E. coli* isolates from the uteri horn, mouth, and rectum of bitches suffering from the disease and 43 *E. coli* isolates from the rectum of clinically healthy bitches were examined for the presence of uropathogenic virulence genes and susceptibility to antimicrobial drugs. DNA profiles of isolates from uteri horn and mouth in bitches with pyometra were compared by REP, ERIC, and BOX-PCR. Virulence gene frequencies detected in isolates from canine pyometra were as follows: 95.7% *fim*, 27.1% *iss*, 25.7% *hly*, 18.5% *iuc*, and 17.1% *usp*. Predominant resistance was determined for cephalothin, ampicillin, and nalidixic acid among the isolates from all sites examined. Multidrug resistance was found on **∼**50% pyometra isolates. Using the genotypic methods some isolates from uteri, pus, and saliva of the same bitch proved to have identical DNA profiles which is a reason for concern due to the close relationship between household pets and humans.

## 1. Introduction

Pyometra (chronic uterine inflammation with accumulation of pus in the uteri) is one of the most common illnesses in bitches [1]. It is a potentially life-threatening condition seen predominantly in middle-aged to aged bitches. The disease normally occurs in the luteal phase of the oestrous cycle and it is associated with hormonal alterations and bacterial infections [1]. Clinical signs vary depending on the stage of the disease, but common manifestations are vaginal discharge, polydipsia, polyuria, inappetence, and lethargy. The safest treatment of pyometra is surgical ovariohysterectomy (immediate removal of the source of infection), which also prevents recurrence [2, 3].

Sometimes* Streptococcus* species,* Klebsiella* species,* Staphylococcus* species,* Pasteurella* species,* Proteus* species, and* Pseudomonas* species are isolated from pyometra infections [4], but* Escherichia coli* are isolated from the uterine contents in the majority (82–100%) of clinical cases of pyometra, and the strains involved in pyometra displayed great similarity with* E. coli* isolates obtained from urinary tract infections (UTI), probably because both of these clinical manifestations arise as bacterial infections and ascend from the host's vaginal or intestinal flora [4]. It has been demonstrated that the epithelium and endometrium of the urinary tract have affinity to* E. coli* when sensitized by a high level of progesterone [5, 6]. Uropathogenic* E. coli* (UPEC) differs from commensal intestinal* E. coli* due to the presence of specialized virulence factors (VF) such as adherence and iron uptake systems as well as cytotoxins and uropathogenic specific protein (*usp*) [7, 8].

Dogs represent potential spreading sources of antimicrobial resistance due to the extensive use of antimicrobial agents in these animals and their close contact with humans [9]. Already, several studies suggest a possible exchange of resistant organisms and/or their resistance genes between humans and their pets [9, 10]. There have been a few studies of resistance to antimicrobials among bacteria isolated from the uteri of bitches with pyometra [11–13].

Pyometra is one of the main diseases in the bitch, and it represents a potential risk to public health since vaginal secretion can be a source of infection to humans and the majority of* E. coli* strains isolated from uteri and urinary tract of bitches with pyometra are similar [4]. The main purpose of this study was to evaluate the antimicrobial resistance and presence of virulence genes on* E. coli* isolates obtained from different sites in bitches suffering from pyometra and to find out the similarity between the obtained isolates.

## 2. Materials and Methods

### 2.1. Collection of Samples from Pyometra and Healthy Bitches

Twenty-eight clinical cases of pyometra diagnosed at a private clinic in Ituverava city, São Paulo State, were studied from March to August 2012. The diagnosis was based on clinical signs and was usually confirmed by radiography and/or ultrasonography. Surgical ovariohysterectomy was thereafter performed. Immediately after removal of the uteri, using aseptic techniques and sterile discardable syringes and needles, 5 mL samples were taken from each uteri horn. At the same time a sterile cotton swab was collected from the mouth (saliva) and the rectum (feces). At the same clinic in the same period of time a sterile cotton swab was collected from the rectum (feces) of 6 clinically healthy bitches, with almost the same mean age. Fecal strains obtained from healthy control subjects have been used to detect potential uropathogenic virulence factors among pyometra isolates by comparing both of them.

The samples were transported to the laboratory in refrigerated conditions. Within 1 hour after collection all specimens were inoculated onto blood agar (Oxoid) and MacConkey agar (Oxoid) at 37°C for 24 h. The isolated microorganisms were identified [15] and classified according to Krieg and Holt [16]. All bacterial strains were stored at −20°C. Only* E. coli* isolates were studied in the present work. From the original MacConkey plates at least five colonies from each site of the collection in the bitches with pyometra and at least 10 colonies from the feces of the clinically healthy bitches were randomly chosen, subcultured on MacConkey agar, and submitted to biochemical tests to be confirmed as* E*.* coli* isolates [17].

### 2.2. Antimicrobial Susceptibility of the* E. coli* Isolates

Antimicrobial disk susceptibility tests were performed using the disk diffusion method as recommended by the Clinical and Laboratory Standards Institute [18] and a total of 12 commercially prepared antimicrobial sensitivity discs (Laborclin, Paraná, Brazil) having the following antimicrobial agents and concentrations were used: amikacin (30 *μ*g), ampicillin (10 *μ*g), cephalothin (30 *μ*g), cefoxitin (30 *μ*g), ceftriaxone (30 *μ*g), ciprofloxacin (5 *μ*g), gentamicin (10 *μ*g), nalidixic acid (30 *μ*g), nitrofurantoin (300 *μ*g), norfloxacin (30 *μ*g), tetracycline (30 *μ*g), and tobramycin (10 *μ*g).* E. coli* reference strains ATCC 25922 and ATCC 35218 were used as control.

### 2.3. Virulence Genes Detection by PCR

*E. coli* strains were grown in Luria Bertani broth (LB-Oxoid) at 37°C overnight (16 h). Bacteria were pelleted from 1.5 mL broth, suspended in 200 *μ*L sterile distilled water, and boiled at 100°C for 10 min. Following centrifugation of the lysate, a 150 *μ*L sample of the supernatant was stored at –20°C as a template DNA stock [19]. PCR assays to detect the* pap*GII (encoding* pap*GII adhesin in P fimbriae),* fim* H (type 1 fimbriae),* hly* F (*α*-haemolysin),* iuc* D (aerobactin),* usp* (uropathogenic specific protein), and* iss* (increased serum survive) were performed on all* E. coli* isolates using established primer pairs [8, 14]. Predicted sizes of the amplified products and specific annealing temperatures are given in Table 1.

PCR amplification using bacterial DNA extracts as templates was carried out in a total volume of 20.0 *μ*L, containing 4.0 *μ*L of DNA supernatant, 0.8 *μ*L (40 mM) of each primer, 0.4 *μ*L (2 mM) of the four deoxynucleotides triphosphates (dNTPs), 3.6 *μ*L of 10x PCR buffer (100 mM Tris-HCl (pH 8.8), 15 mM MgCl_2_, 500 mM KCl, 1% Triton X-100), and 0.2 *μ*L Taq DNA polymerase (Fermentas). Amplification procedure consisted in initial denaturation at 94°C for 5 min in an Eppendorf Mastercycle Gradient thermocycler, followed by 25 denaturation cycles at 94°C for 30 sec, annealing at specific temperatures for 45 sec, extension at 72°C for 1 min, and a final extension step at 72°C for 7 min.

Five microliters of buffer solution (0.25% bromophenol blue in 50% glycerol) were added to each reaction mixture and 10 *μ*L of the final solution was analyzed by electrophoresis on 2% agarose gels (Amersham Pharmacia Biotech) in Tris acetate buffer for 40 min using a Gibco BRL horizontal gel electrophoresis apparatus. Amplicons were stained with ethidium bromide (1.5 *μ*g/mL) for 15 min and gels were photographed. Sizes of the amplicons were determined by comparison with the 1 Kb DNA ladder (Fermentas) run on the same gel.* E. coli* strains FVL2, FVL8, and FVL16 were provided by Dr. D.S. Leite, Unicamp, Campinas, and the strain Ecl 13256 was provided by Dr. Fairbrother, J.M, Faculté de Médecine Vétérinaire de L' Université de Montréal for the FCAV, Veterinary Pathology Department. They were used as a virulence gene control.

### 2.4. Genotypic Methods

Genomic DNA was isolated from the* E. coli* isolates using a Wizard Genomic DNA Purification Kit (Promega), following the manufacturer's instructions. The genetic diversity was evaluated using REP-PCR primers: Rep1R-I (5′ III ICG ICG ICA TCI GGC 3′) and Rep2-I (5′-ICG ICT TAT CIG GCC TAC-3′), ERIC-PCR primers: ERIC 1R (5′ ATG TAA GCT CCT GGG GAT TCA C 3′) and ERIC 2 (5′ AAG TAA GTG ACT GGG GTG AGC G 3′), and BOX A1R primer (5′ CTA CGG CAA GGC GAC GCT GAC G 3′) [20–22].

One PCR mixture was used for all amplifications: (reaction volume 20 *μ*L), buffer 1X (100 mM Tris-HCl pH 8.8; 500 mM KCl; 0.8% (v/v) Nonidet P40); 2 mM MgCl_2_; 0.2 mM dNTPs; 1.5 U Taq polymerase; 5 *ρ*mol each primer; 60 ng genomic DNA; and double distilled water till 20.0 *μ*L. PCR program consisted of the following steps: 4 min at 94°C, 35 cycles of 1 min at 94°C, 1 min at 40°C for REP-PCR, 50°C for ERIC-PCR; 55°C for BOX-PCR, 1,5 min at 72°C, and a final step of 10 min at 72°C. After PCR, the product was loaded on a 1% agarose gel and photographed using a Gel DOC XR photodocumentation system (Bio-Rad). Amplicons sizes were determined by comparison with a 100 pb DNA ladder (Invitrogen).

To perform REP, ERIC, and BOX fingerprint analyses, a binary matrix compounded by one and zero, representing presence and absence of band, respectively, was converted in a distance matrix with the software PAUP version 4.0 b10 (phylogenetic analysis using parsimony) [23]. A dendrogram of the strains was visualized using UPGMA (unweighted pair group method using arithmetic average) grouping using the software MEGA 4.0 (Molecular Evolutionary Genetics Analysis) [24]. The same software was used to evaluate the genetic distance among the isolates.

## 3. Results

A total of 84 samples were collected from three sites (uterine horn (pus), mouth (saliva), and rectum (feces)) from the 28 bitches with pyometra diagnosis after the surgical ovariohysterectomy. A bacterial growth was observed in 57 samples (67.8%).* E. coli* was isolated from uterine samples in 9 bitches (32.1%) and among them 6 bitches showed growth from at least two different collections sites (one of them was always from uterine horn). From these selected 6 bitches with pyometra a total of 70* E. coli* isolates were recovered (25 from uteri, 26 from saliva, and 19 from feces) (Table 2) and used for PCR analyses to detect the presence of virulence genes (Table 3). Using* E. coli* isolates collected in different sites of the same animal it is possible to compare the DNA similarity between the isolates. Although all the* E. coli* isolates from bitches with pyometra originated from only six bitches, this is the same number of animals examined by Hagman and Kühn [4]. To permit a comparison, also 43* E. coli* isolates from feces of 6 clinically healthy bitches were analyzed; the results are summarized in Table 3. Sixty-seven isolates (95.7%) were positive for* fim*, 13 isolates (18.5%) were positive for* iuc*, 18 isolates (25.7%) were positive for* hly*, 12 isolates (17.7%) were positive for* usp,* and 19 isolates (27.1%) were positive for* iss* among the canine pyometra isolates. None of the samples analyzed were positive for* papG*II. Of the 70 samples, 2 (2.8%) did not present positivity for any of the VF genes analyzed.

VF genes were present in* E. coli* isolates from both bitches with pyometra (pus, saliva, and feces) and healthy ones (feces) (Table 3). The number of VF genes per isolate is shown in Table 4. Most of the isolates carried only 1 VF gene without any kind of prevalence among the different sites of sample collection.

The susceptibility to 12 antimicrobial agents for the* E. coli* isolates from canine pyometra is shown in Table 5. Among the isolates from uteri pus the highest resistance was observed against cephalothin (68.0%), followed by ampicillin (56.0%) and nalidixic acid (56.0%) while among the isolates from saliva the highest frequencies were for tetracycline (84.6%), ampicillin (84.6%), and nalidixic acid (84.6%) and among the isolates from feces were for nitrofurantoin (78.9%), cephalothin (68.4%), and tetracycline (63.1%). To better understand the susceptibility to antimicrobial agents of the* E. coli* commensal strains from bitches with pyometra and clinically healthy bitches, a table was built using eight antimicrobial agents extensively used in Brazilian veterinary clinics. In the same table is presented a distribution of the number of resistant phenotypes among the* E. coli* isolates (Table 6).

Except for ampicillin, all others antimicrobial agents showed a higher prevalence of resistant phenotype among the* E. coli* isolates from canine pyometra than from healthy dogs. Multidrug resistance (MDR) characterized as resistance to three or more antimicrobial agents was found among the* E. coli* isolates from feces of both bitches, with pyometra and healthy ones, but it was bigger from pyometra (10/19-52.5%) than from healthy ones (8/43-18.5%) (Table 6).

**Figure 1:**
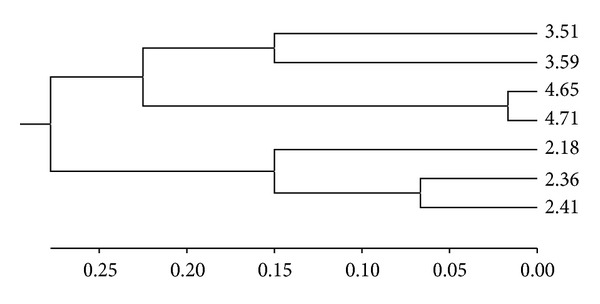
Dendrogram constructed by UPGMA method using genetic distances obtained by REP-PCR analysis of* E. coli* isolates from bitches suffering pyometra: 2/18-bitch 2 isolate 18 from uteri pus; 2/36-bitch 2 isolate 36 from saliva; 2/41-bitch 2 isolate 41 from saliva; 3/51-bitch 3 isolate 51 from saliva; 3/59-bitch 3 isolate 59 from uteri pus; 4/65-bitch 4 isolate 65 from saliva; 4/71-bitch 4 isolate 71 from uteri pus.

*E. coli* strains from different sites in the bitches with pyometra were analyzed by REP, ERIC, and BOX-PCR in order to assess their genetic variability and to discriminate them according to their host. The isolates selected to be analyzed were chosen due to the similarity of the virulence genes encoded and the antimicrobial resistance pattern exhibited by the strains from different sites in each bitch. The results obtained revealed a great genetic diversity among isolates of different animals, but bacteria from the same animal isolated from saliva or the uteri pus display great similarity in some cases. The isolates were grouped in the dendrogram according to the animal from which they were obtained (Figure 1). The greatest genetic diversity was found among isolates from the bitch number 3 (genetic distance: 0.300) in comparison with those from bitch number 2 (genetic distance: 0.244) and 4 (genetic distance: 0.033). The ERIC and BOX-PCR exhibited the same result. The* E. coli* isolates from feces of healthy dogs were not analyzed by REP, ERIC, or BOX-PCR due to the small chance of showing any correlation with samples from bitches with pyometra.

## 4. Discussion

Pyometra is a potentially life-threatening condition seen predominantly in middle-aged to aged bitches and is characterized by the accumulation of pus in the uteri lumen. Up to 24.0% of intact bitches are affected before 10 years of age [25]. In canine pyometra, the uteri are believed to become infected via ascent of bacteria from the vagina through the cervix [2, 26].

Uropathogenic* E. coli* (UPEC) is the pathogen most commonly isolated from canine uteri with pyometra [7, 8, 11]. The ability of* E. coli* to adhere to specific antigenic sites in the endometrium stimulated by progesterone explains the higher prevalence of* E. coli* in pyometra [1, 6].

Bacterial attachment to mucosal membranes, the first step in infection, is commonly facilitated by adhesins, which bind to glycoconjugate receptors on the mucosal surface [6, 27]. The* fim* H adhesin gene can be detected in 100% of* E. coli* pyometra isolates from bitches by PCR [7] and has been demonstrated to facilitates binding of UPEC to canine endometrium [6]; in the present study this gene has been detected around 100% of the isolates, not only among those from uteri pus but also among those from saliva and feces from canine pyometra as well as from feces of healthy bitches (Table 3).

Other uropathogenic virulence factors (UVF) are also important to explain the* E*.* coli* pathogenesis in pyometra. The* pap*G gene has been reported in dogs [8, 11, 28, 29]. There are three variants of this molecule (Class I, II, and III). Allele I is predominantly detected in feces isolates, allele II in pyelonephritis isolates, and allele III in acute cystitis isolates [30, 31].* pap*GII is usually associated with pyelonephritis and was observed in 22.0% of the strains isolated from canine UTI [29]; however, this gene was not detected in the UTI isolates reported by Siqueira et al. [8], but the author reported 5.8% of the* pap*GII gene among the isolates from pyometra. Therefore, the presence of* pap*GII in the* E. coli* isolates obtained from uterine samples may support the fact that pyelonephritis is one of the most serious complications of pyometra; so, it is important to search for* pap*GII gene. In this study* pap*GII has not been detected among all the isolates examined from different sites.

In addition to* pap*, the canine strains contained other virulence-associated genes characteristic of human ExPEC strains. *α*-Haemolysin is a pore-forming toxin that lyses not only erythrocytes [30] but also leukocytes, endothelial, and renal epithelial cells [32]. It has been reported in a great number among* E. coli* isolates from canine pyometra, as reported 52.0% [7], 34.4% [11], and 34.6% [8], while it was reported in a small number among the* E. coli* isolates from feces 25.0% [7] and 12.7% [8]. In the present study* hly* gene was found in the opposite distribution, 16.0% in pyometra (pus) isolates, and 47.3% in feces isolates from the same bitch. However, when the focus was in the prevalence of* hly* gene among the* E. coli* isolates from the feces of healthy bitches, the frequency of 16.2% was close to those reported by others [7, 8] which always compare isolates from pyometra (uteri pus) with isolates from feces of healthy bitches.

Almost the same situation was found for two other virulence genes when compared with the results reported by Siqueira et al. [8]. The* iuc* D (iron uptake chelate) gene encoded an enzyme involved in the biogenesis of aerobactin [30]. It was found in Siqueira et al. [8] in 17.3% of* E. coli* isolates from pyometra and in 1.8% of isolates from feces, while in the present study it was found in 4.0% of the isolates from pyometra (pus) and 36.8% in isolates from feces from the same bitch. The* usp* (uropathogenic specific protein) gene was reported among* E. coli* isolates from urine and feces of companion animals (cats and dogs) [33] and it is supposed to act as a bacteriocin. Siqueira et al. [8] reported the prevalence of 69.2% in* E. coli* isolates from pyometra and 0% in isolates from feces. In the present study the prevalence of 20.0% was found among the* E. coli* isolates from pyometra and 15.7% in isolates from feces.

Taken together the results reported by Chen et al. [7] and Siqueira et al. [8] showed that* E. coli* isolates from pyometra (pus) carried some VF genes (*hly* A,* iuc* D, and* usp*) in a higher percentage than in the* E. coli* isolates from feces of healthy bitches used as a control. Chen et al. [7] demonstrated a linkage of VF genes with the* E. coli* isolates from canine pyometra, with a great number of VF genes per isolate. In the present work the results obtained do not indicate this predominance of VF genes among the pyometra isolates and most of isolates from all the sites examined, except for two isolates one from pyometra and one from feces, carry one or two VF genes per isolate.

The increased serum survival gene (*iss*) has long been recognized for its role in ExPEC virulence;* iss* has been identified as a distinguishing trait of avian ExPEC but not of human ExPEC [34]. However, recently the* iss* gene was reported by Huber et al. [35] in a dog strain associated with extended spectrum *β*-lactamase- (ESBL-) producing* E. coli*. The exact importance of this gene is not well known, so it has been tested in all the* E. coli* strains recovered in the present study. It observed a very similar frequency between the* E. coli* isolates from uteri pus (12.0%) and saliva (19.2%) but a very different frequency from the isolates from feces (57.8%) in the canine pyometra. At the moment we do not have an explanation for the discrepancies above mentioned.

The relationship between companion animals and humans has changed radically throughout the years, with dogs being more and more in close contact with humans. Close physical contact by touching, petting, and licking occurs at high frequency on the basis of the current perception of household pets as actual family members what tremendously increases the risk of a transmission of antimicrobial resistant bacteria from pets to humans [9, 36], or the transmission of other genes involved with virulence factors [34].

The antimicrobial resistances in strains of* E. coli* from pyometra have been reported by several authors [11–13]. The highest resistance among the* E. coli* pyometra isolates in this study was to cephalothin and ampicillin agreeing with Coggan et al. [11] but higher than the results reported by Siqueira et al. [12] and Inoue et al. [13] to ampicillin. Also the results reported to ciprofloxacin, norfloxacin, and gentamicin sensitivity agree with those reported by Siqueira et al. [12], Coggan et al. [11], and Inoue et al. [13].

In the present study for the first time the antimicrobial resistance of* E. coli* isolates from feces of bitches suffering pyometra was compared with the resistance of isolates from healthy bitches. The high resistance frequencies found among the isolates from pyometra bitches could suggest that the bitches with pyometra have been in contact with antimicrobial drugs for a long time and have been selecting microorganisms with a great number of resistant genes which is a reason for concern due to the close contact with humans.

An elevated degree of multiresistance among the* E. coli* isolates is a great problem because canine MDR* E. coli* has been shown to possess class I integron-associated resistance genes that have previously been identified in bacterial isolates from clinical infections in humans [10]. This suggests the spread of common resistance mechanisms between canine and human bacterial isolates [37].

The present study showed that the DNA profiles of* E. coli* isolates from pyometra infections in different bitches varied greatly and thus belonged to different clones, a finding that supports the results of Wadås et al. [26] and Chen et al. [7]. The* E. coli* isolates from uteri pus and saliva of the same animal were sometimes indistinguishable as shown in this work, which is a reason for concern due to the close relationships between household pets and humans. Studies are underway in our laboratory to verify for how long the* E. coli* strains in saliva are viable and could be transmitted.

## Figures and Tables

**Table 1:** PCR: genes, primer sequences, and amplicons size.

Genes	Primer sequence (5′–3′)	Size (bp)	Reference
*fim* H	TGCAGAACGGATAAGCCGTGG GCAGTCACCTGCCCTCCGGTA	508	Siqueira et al., 2009 [8]

*iuc* D	TACCGGATTGTCATATGCAGACCGT AATATCTTCCTCCAGTCCGGAGAAG	602	Siqueira et al., 2009 [8]

*hly* F	GGCCACAGTCGTTTAGGGTGCTTACC GGCGGTTTAGGCATTCCGATACTCAG	450	Johnson et al., 2008 [14]

*usp *	ATGCTACTGTTTCCGGGTAGTGTGT CATCATGTAGTCGGGGCGTAACAAT	1000	Siqueira et al., 2009 [8]

*iss *	CAGCAACCCGAACCACTTGATG AGCATTGCCAGAGCGGCAGAA	323	Johnson et al., 2008 [14]

*papGII *	GGAATGTGGTGATTACTCAAAGG TCCAGAGACTGTTCAAGAAGGAC	562	Siqueira et al., 2009 [8]

**Table 2:** Number of *E. coli* isolates obtained from different sampling sites of bitches with pyometra.

Bitch	Sites of sample collection		
	Uteri pus (*n* = 25)	Saliva (*n* = 26)	Feces (*n* = 19)
1	3*	5	5
2	5	5	5
3	5	5	5
4	5	5	0
5	4	5	0
6	3	1	4

*Number of *E. coli* isolates.

**Table 3:** Prevalence of genes encoding virulence factors in *E. coli* isolates from uteri horn (pus), saliva, and feces from 6 bitches suffering pyometra and from feces of 6 clinically healthy bitches.

Gene	Bitches with pyometra			Healthy bitches
	Uteri pus (*n* = 25)	Saliva (*n* = 26)	Feces (*n* = 19)	Feces (*n* = 43)
*fim* H	23 (92.0%)	26 (100.0%)	18 (94.7%)	41 (95.3%)
*iuc* D	1 (4.0%)	5 (19.2%)	7 (36.8%)	9 (20.9%)
*hly* A	4 (16.0%)	5 (19.2%)	9 (47.3%)	7 (16.2%)
*usp *	5 (20.0%)	4 (15.3%)	3 (15.7%)	9 (20.9%)
*iss *	3 (12.0%)	5 (19.2%)	11 (57.8%)	6 (13.9%)

**Table 4:** Linkage of virulence genes in isolates of *E. coli* from canine pyometra and feces of healthy bitches.

Number of virulence genes per isolate	Number of *E. coli* isolates			
	Bitches with pyometra			Healthy bitches
	Uteri pus	Saliva	Feces	Feces
None	1	0	1	0
1	16	15	7	23
2	6	5	1	13
3	1	4	3	5
4	0	2	5	2
5	1	0	2	0

**Table 5:** Prevalence of antimicrobial resistance among 70 *E. coli* isolates from 6 bitches suffering pyometra.

Antimicrobial drug	Uteri (pus) (*n* = 25)	Mouth (saliva) (*n* = 26)	Rectum (feces) (*n* = 19)
Norfloxacin	0* (0.0%)**	5 (19.2%)	1 (5.2%)
Cefoxitin	0 (0.0%)	0 (0.0%)	0 (0.0%)
Tobramycin	0 (0.0%)	7 (26.9%)	6 (31.5%)
Ciprofloxacin	2 (8.0%)	1 (3.8%)	2 (10.5%)
Gentamicin	3 (12.0%)	6 (23.0%)	5 (26.3%)
Ceftriaxone	5 (20.0%)	5 (19.2%)	7 (36.8%)
Tetracycline	8 (32.0%)	22 (84.6%)	12 (63.1%)
Amikacin	10 (40.0%)	10 (38.4%)	11 (57.8%)
Nitrofurantoin	10 (40.0%)	16 (61.5%)	15 (78.9%)
Ampicillin	14 (56.0%)	22 (84.6%)	9 (47.3%)
Nalidixic acid	14 (56.0%)	22 (84.6%)	8 (42.1%)
Cephalothin	17 (68.0%)	17 (65.3%)	13 (68.4%)

*Number of isolates; **percentage.

**Table 6:** Antimicrobial resistance (percentage) and distribution of number of resistant phenotypes among the *E. coli* commensal isolates from feces of bitches with pyometra and healthy bitches.

Antimicrobial drugs	Feces	
	Bitches with Pyometra (*n* = 19)	Healthy bitches (*n* = 43)
Ampicillin	47.3%	41.1%
Cephalothin	68.4%	35.2%
Ceftriaxone	36.8%	0.0%
Tetracycline	63.1%	41.1%
Gentamycin	26.3%	11.7%
Amikacin	57.8%	17.6%
Nalidixic acid	42.1%	17.6%
Ciprofloxacin	10.5%	0.0%

Number of resistant phenotypes	Number of *E. coli* isolates	

0	2	19
1	3	8
2	4	8
3	2	3
4	1	3
5	4	2
6	2	0
7	1	0
